# A Quality Improvement Project to Evaluate the Utilisation of Different Visual/Auditory/Kinaesthetic Techniques to Support Learning Within the Local MRCPsych Programme

**DOI:** 10.1192/bjo.2026.11320

**Published:** 2026-06-30

**Authors:** Jasmine Fox, Unsa Athar, Indira Vinjamuri

**Affiliations:** Mersey Care, Liverpool, United Kingdom

## Abstract

**Aims:**

The weekly Local Academic Programme (LAP) at Mersey Care NHS Foundation Trust is part of the MRCPsych programme. It aims to prepare psychiatry trainees for theirMRCPsych exams, and within Mersey Care receives positive feedback from attending resident doctors. To support exam practice, multiple-choice questions (MCQs) were introduced in 2024. We decided to extend further support as trainee leads and introduced didactic (maximum 5 minutes) explanations of complex exam topics every session, using different audio/visual technologies. The aim was to cater to different learning methods; Visual, Audio, Reading/Writing, Kinaesthetics.

**Methods:**

The quality improvement project used mixed methodology. We used convenience sampling to collect quantitative and qualitative data. Before the start of the semester, a pre-intervention survey was sent to trainee doctors using Google Forms, in February 2025. After 5 MCQs for the audience to practice in the LAP, one of the authors (LAP trainee leads) explained a chosen topic for 5 minutes. We used power point presentations or videos using graphics or white board function on Zoom to explain examinable topics. Paper A and Paper B curriculums were used to select the topic for discussion. Throughout the semester and at the end, we sent out surveys to gather feedback using Smart Surveys until July 2025.

**Results:**

Initial survey data indicated that most trainees struggled to digest concepts in standard MCQ banks and believed they would benefit from short (5-minute) discussions on complex examinable topics, such as biostatistics (e.g. ANOVA vs. ANCOVA) during our sessions.

Following the implementation of the new LAP format, 60.7% found MCQ based explanations helpful. Regarding delivery style, 35% of respondents preferred a mixture of MCQs and whiteboard/video explanations, while 33.33% favoured MCQs with standard explanations. None of the respondents wanted whiteboard/video-based teaching alone.

Qualitative feedback was highly positive, with trainees noting that the explanation of statistical methods was “particularly good” and “very helpful for revision” for Paper B. Participants specifically praised the “simplified” explanations to consolidate learning.

**Conclusion:**

The trainees found the new sessions useful for their organisation, interactivity, and clarity on difficult to understand examinable topics. To support the greatest benefit in MRCPsych examination preparation, trainees preferred a combined approach of MCQ style questions alongside whiteboard/video explanations for more complex topics. Catering to different learning methods and differential attainment in local medical education programmes could potentially improve MRCPsych success rates for resident doctors in psychiatry training.

